# Engineering a trifunctional proline utilization A chimaera by fusing a DNA-binding domain to a bifunctional PutA

**DOI:** 10.1042/BSR20160435

**Published:** 2016-11-22

**Authors:** Benjamin W. Arentson, Erin L. Hayes, Weidong Zhu, Harkewal Singh, John J. Tanner, Donald F. Becker

**Affiliations:** * Department of Biochemistry, Redox Biology Center, University of Nebraska-Lincoln, Lincoln, NE 68588, U.S.A.; † Department of Biochemistry, University of Missouri-Columbia, Columbia, MO 65211, U.S.A.; ‡ Department of Chemistry, University of Missouri-Columbia, Columbia, MO 65211, U.S.A.; § Protein Technologies and Assays, Research and Development, MilliporeSigma, 2909 Laclede Avenue, St. Louis, MO 63103, U.S.A.

**Keywords:** DNA binding, flavin enzyme, proline metabolism, small-angle X-ray scattering (SAXS)

## Abstract

Proline utilization A (PutA) is a bifunctional flavoenzyme with proline dehydrogenase (PRODH) and Δ^1^-pyrroline-5-carboxylate (P5C) dehydrogenase (P5CDH) domains that catalyses the two-step oxidation of proline to glutamate. Trifunctional PutAs also have an N-terminal ribbon–helix–helix (RHH) DNA-binding domain and moonlight as autogenous transcriptional repressors of the *put* regulon. A unique property of trifunctional PutA is the ability to switch functions from DNA-bound repressor to membrane-associated enzyme in response to cellular nutritional needs and proline availability. In the present study, we attempt to construct a trifunctional PutA by fusing the RHH domain of *Escherichia coli* PutA (EcRHH) to the bifunctional *Rhodobacter capsulatus* PutA (RcPutA) in order to explore the modular design of functional switching in trifunctional PutAs. The EcRHH–RcPutA chimaera retains the catalytic properties of RcPutA while acquiring the oligomeric state, quaternary structure and DNA-binding properties of EcPutA. Furthermore, the EcRHH–RcPutA chimaera exhibits proline-induced lipid association, which is a fundamental characteristic of functional switching. Unexpectedly, RcPutA lipid binding is also activated by proline, which shows for the first time that bifunctional PutAs exhibit a limited form of functional switching. Altogether, these results suggest that the C-terminal domain (CTD), which is conserved by trifunctional PutAs and certain bifunctional PutAs, is essential for functional switching in trifunctional PutAs.

## INTRODUCTION

**Scheme 1:**
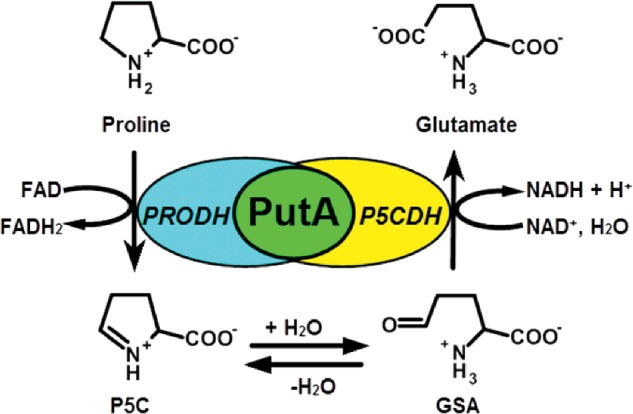
Reactions catalysed by the bifunctional PutA enzyme. The PRODH domain catalyses the oxidation of proline to P5C using a flavin cofactor as the electron acceptor. P5C undergoes a non-enzymatic hydrolysis, resulting in GSA. The P5C dehydrogenase domain (P5CDH) catalyses the NAD^+^-dependent oxidation of GSA to glutamate thereby generating NADH.

The proline catabolic pathway converts proline into glutamate via two consecutive steps catalysed by proline dehydrogenase (PRODH) and Δ^1^-pyrroline-5-carboxylate (P5C) dehydrogenase (P5CDH) (Scheme 1). In the first reaction, PRODH forms the intermediate P5C through the flavin-dependent, two-electron oxidation of proline. P5C then undergoes a non-enzymatic hydrolysis step, forming glutamate-γ-semialdehyde (GSA), which is oxidized to glutamate by NAD^+^-dependent P5CDH. The reactions of proline catabolism affect a broad array of physiological processes in different organisms [1–4] and have underlying roles in human diseases such as cancer [5,6] and schizophrenia [7–9]. Proline is also a critical respiratory substrate for *Helicobacter pylori* during infection [10,11] and in the fungal pathogen, *Cryptococcus neoformans*, proline catabolism was previously shown to be required for virulence in mice [12].

PRODH and P5CDH are separate enzymes in eukaryotes and Gram-positive bacteria, whereas in Gram-negative bacteria the enzymes are encoded as a bifunctional polypeptide known as proline utilization A (PutA) [13,14]. In addition to catalytic activities, a subset of Gram-negative bacteria such as *Escherichia coli* and *Salmonella typhimurium* encode PutAs with a DNA-binding domain, which allows PutA to repress transcription of *putA* and *putP* (proline transporter) genes [15,16]. The DNA-binding domain in trifunctional PutA provides a unique coupling between proline availability and proline metabolic gene expression [15,16].

In *S. typhimurium* and *E. coli*, trifunctional PutA switches from a DNA-bound transcriptional repressor to a membrane-bound enzyme based on intracellular proline levels and the redox state of the flavin cofactor [16–18]. This change in intracellular location and molecular function, known as functional switching, enables bacteria to utilize environmental proline as a fuel source [19]. Proline reduction of the flavin cofactor significantly increases PutA membrane binding affinity while diminishing PutA–DNA binding affinity by only 2-fold [18,20–23]. The DNA-binding domain of trifunctional PutAs is located at the N-terminus and has been shown by X-ray crystallography to have the ribbon–helix–helix (RHH) fold [15,24,25]. In contrast, the location of the membrane-binding domain of PutA is an active area of research. Previous studies of *E. coli* PutA (EcPutA) have identified conformational changes in the flavin cofactor and active site residues of the PRODH domain that are critical for mediating redox activation of PutA membrane interactions [19,26,27]. Additionally, proline-dependent conformational changes occur outside the PRODH domain that correlate with increased PutA membrane binding [21]. These studies implicate a helical domain near the PRODH active site in mediating membrane association, but how conformational changes in PutA enhance membrane interactions remains unclear.

The PutA protein family comprises three basic domain architectures [28,29]. Type A PutAs are approximately 1000 residues in length and have the minimal set of domains needed for the two catalytic activities. The crystal structures of the type A PutAs from *Bradyrhizobium japonicum* [30] and *Geobacter sulfurreducens* [31] have revealed the structural basis of PutA catalytic activity including a tunnel between the PRODH and P5CDH domains for channelling the P5C/GSA intermediate. Sequence analysis suggests that the catalytic core observed in these structures is highly conserved throughout the entire PutA family. Type B PutAs are larger (1100–1200 residues) and have a 100–200 C-terminal domain (CTD) in addition to the catalytic core. We recently showed that the CTD of the type B PutA from *Rhodobacter capsulatus* (RcPutA) contributes to aldehyde dehydrogenase activity and substrate channelling [29]. Type C PutAs (approximately 1300 residues) have both the CTD and the RHH domain; all trifunctional PutAs have the type C architecture.

**Figure 1:**
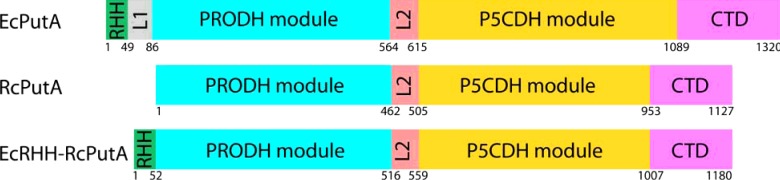
Domain organization maps of EcPutA, *R. capsulatus* PutA (RcPutA) and EcRHH–RcPutA In EcRHH–RcPutA, the RHH DNA-binding domain of EcPutA was fused to the N-terminus of *R. capsulatus* PutA. Numbers below the domain map indicate amino acid position. L1 and L2 indicate inter-domain linkers.

The aim of the present study was to investigate the modularity of PutA domain architectures by converting a type B PutA into a type C PutA using protein engineering. The RHH DNA-binding domain of EcPutA (EcRHH) was fused to the N-terminus of RcPutA to create the chimaeric protein EcRHH–RcPutA (Figure 1). RcPutA and EcPutA are 47% identical (61% similar), making RcPutA a suitable candidate for studying the effects of adding an N-terminal RHH domain. We show that the addition of the EcRHH domain to RcPutA generates a chimaeric protein that resembles trifunctional EcPutA in terms of oligomeric state, quaternary structure, DNA-binding and proline-dependent lipid association.

## MATERIALS AND METHODS

### Materials

Unless noted, all chemicals were purchased from Sigma–Aldrich or Thermo Fisher Scientific. Nanopure water was used in all experiments.

### Expression constructs and protein purification

A chimaera consisting of the RHH domain of EcPutA (EcRHH, residues 1–52) fused to the N-terminus of RcPutA was engineered. The EcRHH–RcPutA enzyme construct was made by PCR amplification of the DNA-binding domain (residues 1–52) of EcPutA using a pET14b-EcPutA construct described previously [32]. *NdeI* restriction sites were incorporated at both ends of the PCR product using primers 5′-CGGCGCCATATGATGACCGACCTTTCCGCCCTTGG-3′ and 5′-CGCCGCCATATGCTCCGGCAGAGTATCGCTGT-TTTCC-3′. The PCR product was then inserted into the previously made pET28a-RcPutA [29] construct immediately upstream of RcPutA gene (*NdeI*). The resulting pET28-EcRHH-RcPutA construct contained an amino acid linker between EcRHH and RcPutA and was confirmed by DNA sequencing (Eurofins MWG Operon).

EcPutA was overexpressed and purified as described previously [22,33]. RcPutA (1127 amino acids) and EcRHH–RcPutA (1180 amino acids), both in a pET28a vector, were overexpressed in BL21(DE3) pLysS cells (Promega). Starter cultures (5 ml) were grown overnight in LB medium (25 μg/ml kanamycin, 34 μg/ml chloramphenicol) and used to inoculate 4 litre cultures of LB medium plus antibiotics. Once cultures reached absorbance at 600 nm (*A*_600_) of 0.8, 0.5 mM IPTG was added, and cultures were grown overnight at 20°C before harvesting by centrifugation at 16 800 ***g*** for 30 min and freezing the cell pellets at −80°C.

The frozen cell pellets were resuspended in 50 ml binding buffer (20 mM Tris-base, 0.5 M NaCl, 5 mM imidazole, 10% glycerol, pH 7.9) at 4°C containing FAD (0.1 mM) and protease inhibitors ε-amino-*N*-caproic acid (3 mM), phenylmethylsulfonyl fluoride (0.3 mM), leupeptin (1 μM), tosyl phenylalanyl chloromethyl ketone (48 μM) and tosyllysine chloromethyl ketone hydrochloride (78 μM). Cells were disrupted by sonication at 4°C. The solubility of the proteins was optimized by adding 0.25 mM Triton X-100 to the cell lysate and incubating at 4°C with slow stirring for 30 min. The cell lysate was centrifuged for 1 h at 45 980 ***g*** in a JA-20 rotor (Beckman). The supernatant was then filtered (0.2 μm filter, VWR) and loaded on to a Ni-NTA Superflow affinity column (Qiagen) equilibrated with Tris-base binding buffer (pH 7.9). Wash buffer (60 mM imidazole) followed by elution buffer (500 mM imidazole) were then applied to the column. Elution fractions containing PutA protein were dialysed overnight at 4°C into 50 mM Tris (pH 7.5) buffer containing 10 mM NaCl, 0.5 mM EDTA and 10% glycerol. The protein was then loaded on to an anion exchange column (HiTrap Q HP column, GE Life Sciences) equilibrated with the Tris buffer above. A linear gradient of 0–1 M NaCl in Tris buffer (pH 7.5, 0.5 mM EDTA, 10% glycerol) was used to elute PutA. Purified RcPutA and EcRHH–RcPutA were then dialysed into 50 mM Tris (pH 7.5) containing 50 mM NaCl, 0.5 mM EDTA and 10% glycerol and stored at −80°C. Protein purity was analysed by SDS/PAGE. Protein concentration was determined by the 660 nm Protein Assay (Thermo Scientific) using BSA as the standard. The amount of bound FAD cofactor in purified RcPutA and EcRHH–RcPutA was determined as previously described [17]. The N-terminal hexahistidine tag was retained after purification.

### PRODH kinetic assays

All steady-state assays were performed at 23°C. Kinetic parameters for proline were determined using coenzyme Q_1_ (CoQ_1_) as an electron acceptor and monitoring CoQ_1_ reduction by the decrease in absorbance at 278 nm (*ε*=14500 M^−1^ cm^−1^) [33]. *K*_m_ and *k*_cat_ for proline were determined for RcPutA and EcRHH–RcPutA (0.25 μM) by varying proline (0–100 mM) and fixing CoQ_1_ at 300 μM. Likewise, the *K*_m_ and *k*_cat_ for CoQ_1_ were determined by varying CoQ_1_ (10–450 μM) while holding proline constant at 200 mM. The above assays were conducted in 50 mM potassium phosphate (pH 7.5) and 25 mM NaCl. Data were collected using a 0.15 cm path length on a Hi-Tech Scientific SF-61DX2 stopped-flow instrument. Kinetic parameters were determined by fitting initial velocities to the Michaelis–Menten equation (SigmaPlot 12.0) [34].

### PRODH–P5CDH coupled assay

PRODH–P5CDH coupled activity, in which proline is converted to glutamate, was measured as described previously [30]. Briefly, 0.25 μM enzyme was mixed with 200 μM CoQ_1_, 40 mM proline and 200 μM NAD^+^ in 50 mM potassium phosphate (pH 7.5) containing 25 mM NaCl. The progress of the reaction was followed by NAD^+^ reduction at 340 nm (*ε*_340_=6200 M^−1^ cm^−1^).

### Oligomeric structure determination

The oligomeric states of RcPutA and EcRHH–RcPutA were determined by gel filtration chromatography and sedimentation equilibrium. The purified proteins were loaded on to a Superdex 200 10/300 GL column (GE Life Sciences) and eluted by FPLC in 50 mM Tris buffer (pH 7.5) containing 100 mM NaCl, 0.5 mM EDTA and 10% glycerol at a flow rate of 0.5 ml/min. The molecular mass (*M*) of each protein was estimated using thyroglobulin (669 kDa), apoferritin (443 kDa), β-amylase (200 kDa), BSA (66 kDa) and carbonic anhydrase (29 kDa) as molecular mass standards.

Sedimentation equilibrium was performed using an Optima XL-I analytical ultracentrifuge (Beckman Coulter) equipped with an eight-hole An50 Ti rotor. EcRHH–RcPutA (2 mg/ml) was dialysed in 50 mM Tris buffer (pH 7.5) containing 100 mM NaCl, 0.5 mM EDTA and 5% glycerol. The dialysed EcRHH–RcPutA protein was then diluted to three concentrations of 0.2, 0.5 and 0.8 mg/ml, and loaded at 110 μl each into the sample cells. The reference cell was loaded (125 μl) with the dialysate buffer. Radial absorbance scans with a spacing of 0.001 cm were collected at 280 nm for each concentration of EcRHH–RcPutA at 22 h and 24 h after equilibration at 8000 rpm. The scans are an average of ten measurements at each radial position. Origin 6.0 software was used to best-fit the data to a single ideal species model using a solvent density of 1.018 g/ml and a partial specific volume of 0.742 ml/g estimated from the EcRHH–RcPutA sequence by Sednterp software.

### SAXS

For SAXS experiments, EcRHH–RcPutA was expressed as described above and then purified according to the following protocol. Frozen cells were thawed in the presence of 1 mM phenylmethylsulfonyl fluoride and 20 mM *N*-octyl-β-D-glucoside at 4°C. The resulting cell paste was sonicated on ice for 1–2 min and centrifuged at 26 892 ***g*** for 45 min using a SS-34 rotor. The supernatant was transferred into new tubes and centrifuged at 32 539 ***g*** for 45 min. The resulting clear supernatant was loaded on to a Ni^2+^ affinity column (HisTrap HP, GE Healthcare) and EcRHH–RcPutA was eluted using 300 mM imidazole. Samples containing the enzyme were pooled and dialysed overnight against 50 mM Tris/HCl, pH 7.5, 50 mM NaCl, 1 mM EDTA, 1 mM tris(3-hydroxypropyl)phosphine (THP) and 50 μM FAD. The dialysed protein was loaded on to an anion exchange column (HiTrap Q, GE Healthcare) and the column was eluted using a linear gradient of 0–1 M NaCl. Based upon the purity of samples as judged by SDS/PAGE, the appropriate samples were combined and dialysed against 100 mM Tris/HCl, 200 mM NaCl, 0.5 mM THP and 1 mM EDTA at pH7.5. The protein was concentrated to approximately 20 mg/ml using a 30000 kDa cutoff membrane; the protein concentration was measured using the Bradford method. Size exclusion chromatography was performed using a Superdex 200 column equilibrated in 50 mM Tris, 200 mM NaCl, 0.5 mM THP at pH7.5. The protein concentration after size exclusion chromatography was in the range 8–12 mg/ml.

SAXS data were collected at beamline 12.3.1 of the Advanced Light Source through the mail-in program [35,36]. The protein was shipped via overnight courier to the beamline in a 96-well plate that was kept 4°C. For each protein fraction, scattering intensities were measured at three nominal protein concentrations. For each protein concentration, exposure times of 0.5, 1.0, 3.0 and 6.0 s were used. Scattering curves collected from the protein samples were corrected for background scattering using intensity data collected from the effluent from size exclusion chromatography.

The SAXS data were processed as follows. A composite scattering curve for each sample was generated with PRIMUS [37] by scaling and merging the high *q* region from the 3.0 s exposure with the low *q* region from the 0.5 s exposure. The scattering curves were multiplied with a concentration factor and overlaid on each other to check for concentration dependent variation of the profile. No substantial concentration effects were observed.

Structural properties were derived from the merged SAXS profile as follows. The radius of gyration (*R*_g_) was determined from Guinier analysis using PRIMUS. GNOM [38] and SCATTER were used to calculate pair distribution functions in order to estimate the maximum particle dimension (*D*_max_) and real space *R*_g_. The *M* was estimated from the SAXS volume of correlation (*V*_c_) [39] as described previously [40]. Shape reconstruction calculations were performed with GASBOR using *D*_max_ of 183 Å (1 Å=0.1 nm) and 2-fold symmetry. Thirty-two independent GASBOR models were generated and then averaged and filtered using DAMAVER [41]. The Situs module pdb2vol was used to convert the averaged, filtered models into volumetric maps [42]. SUPCOMB was used to superimpose dummy atom models [43]. The SAXS curve and GNOM file used for the analysis described here have been deposited in the Small-angle Scattering Biological Data Bank (SASBDB) under the accession code SASDB27 [44].

### DNA-binding

DNA-binding activity was determined by gel mobility shift assays using fluorescently labelled *put* control DNA from *E. coli* as previously described [24]. A dissociation constant of EcRHH–RcPutA with *put* control DNA was determined by best-fit analysis to eqn (1) (Sigma Plot 12), where *n* is the number of binding sites and [L] is total concentration of protein [17].

1 $$\mathrm{Fraction}\;\mathrm{of}\;\mathrm{DNA}\;\mathrm{Bound}=n\left[L\right]/(K_{d}+\left[L\right])$$

### Lipid pull-down assays

Lipid pull-down assays were performed anaerobically under a nitrogen atmosphere in an anaerobic chamber (Belle Technology Glovebox) as described previously using *E. coli* polar lipids (Avanti Polar Lipids) [26]. In these assays, 50 mM proline was used to reduce the flavin cofactor in the PutA proteins (0.3 mg/ml) during the incubation with lipid vesicles.

## RESULTS

### General properties and steady-state kinetic parameters of EcRHH–RcPutA

EcRHH–RcPutA was expressed as a soluble protein in *E. coli* and has a molecular size on SDS/PAGE consistent with the predicted *M* of 126 kDa. The UV–visible spectrum of EcRHH–RcPutA has absorbance maxima at 380 nm and 451 nm showing incorporation of flavin into the protein fold. The flavin absorbance spectrum indicates 92% flavin content. The incorporation of one FAD per protein monomer is consistent with previous and recent studies of RcPutA [29] and other PutAs [17,30,31].

The PRODH steady-state kinetic parameters of EcRHH–RcPutA were determined for comparison to those previously reported for RcPutA (Table 1). The *K*_m_ and *k*_cat_ values for proline as the variable substrate are 5.3 mM and 0.89 s^−1^, which are close to those of RcPutA (5.6 mM and 1.0 s^−1^). The catalytic efficiencies of the chimaera and RcPutA differ by just 6%. Using CoQ_1_ as the variable substrate, the parameters are *K*_m_ of 161 μM and *k*_cat_ of 1.1 s^−1^. These values are within a factor of two of those of RcPutA. These results show that fusing the EcPutA RHH domain on to RcPutA does not significantly affect the PRODH kinetic parameters.

**Table 1:** PRODH kinetic parameters for EcRHH–RcPutA and RcPutA

		Proline			CoQ_1_	
Enzyme	*K*_m_ (mM)	*k*_cat_ (s^−1^)	*k*_cat_/*K*_m_ (M^−1^ s^−1^)	*K*_m_ (μM)	*k*_cat_ (s^−1^)	*k*_cat_/*K*_m_ (M^−1^ s^−1^)
EcRHH–RcPutA	5.3±0.9	0.89±0.1	167.9±5.2	161.0±15.8	1.1±0.1	6832±715
RcPutA	5.6±0.8	1.0±0.1	178.6±30	93.7±19.0	2.0±0.1	21505±4579

Kinetic parameters were determined in 50 mM potassium phosphate, 25 mM NaCl, pH 7.5. Data obtained from Luo et al. [29].

**Figure 2:**
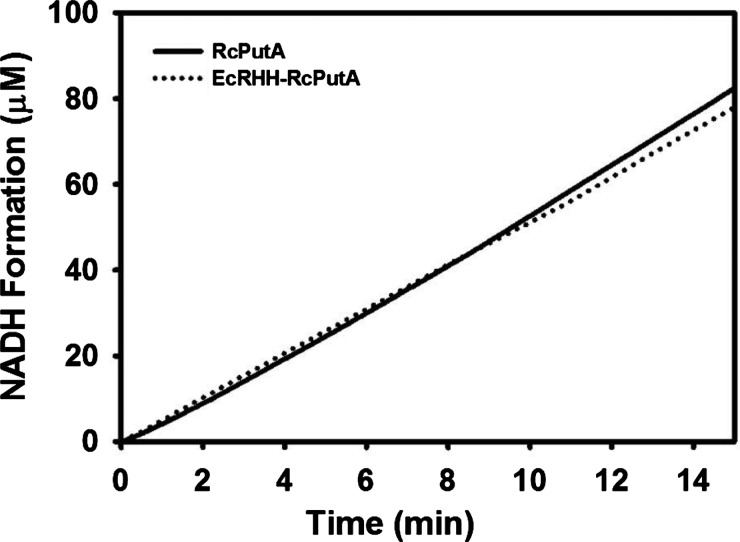
Coupled PRODH–P5CDH activity of EcRHH–RcPutA The assay was performed using 200 μM CoQ_1_, 40 mM proline, 0.25 μM EcRHH–RcPutA in 50 mM potassium phosphate (pH 7.5) and 25 mM NaCl. NADH formation was monitored at 340 nm. For comparison, a trace of RcPutA activity is also shown from a channelling assay performed under identical conditions [29].

Coupled PRODH–P5CDH assays were performed to further explore the kinetic properties of EcRHH–RcPutA. These assays report on the activity of both the PRODH and P5CDH active sites by monitoring NADH formation, which is used as a read out of the overall conversion of proline to glutamate. Figure 2 shows the results of PRODH–P5CDH coupled assays for EcRHH–RcPutA and RcPutA conducted under identical conditions. The steady-state velocity of NADH formation for EcRHH–RcPutA is similar to that of RcPutA, thus providing additional evidence that the fusion of the EcPutA DNA-binding domain does not disrupt the overall kinetic properties of RcPutA. In addition, no lag phase is apparent in these assays suggesting that the intermediate L-P5C/GSA does not equilibrate with bulk solvent but instead is channelled between the PRODH and P5CDH active sites [30]. These results are consistent with that previously described for other PutAs [29–31,45].

### Oligomeric state determination

The domain organization of RcPutA provides an excellent opportunity to explore the impact of the RHH and CTDs on the oligomeric organization of PutAs. Previous studies have shown that the RHH domain mediates dimerization of EcPutA [46]. In contrast, RcPutA is monomeric [29], which is consistent with RcPutA lacking the RHH domain of trifunctional PutA. The oligomeric state of EcRHH–RcPutA was therefore determined to see whether fusion of the RHH domain converts RcPutA into a dimeric protein.

**Figure 3:**
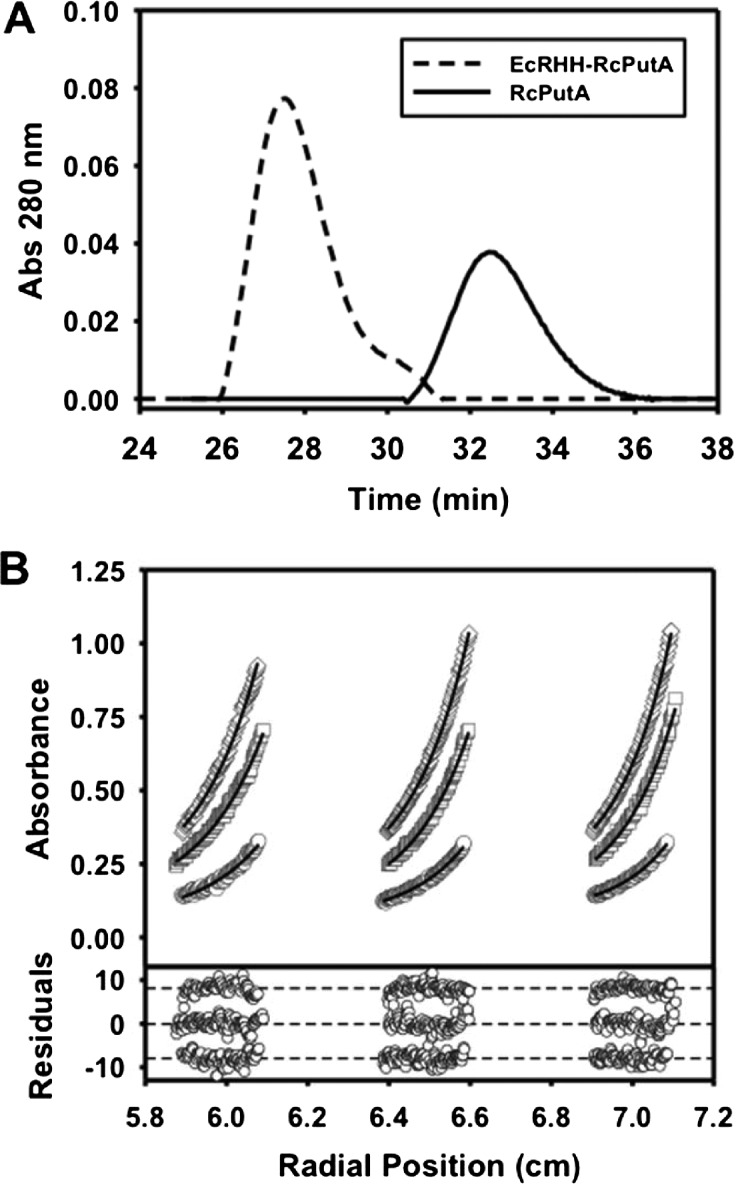
Oligomeric structure analysis of EcRHH–RcPutA (**A**) Gel filtration elution profiles (monitored at 280 nm) of RcPutA (solid trace) and EcRHH–RcPutA (dotted trace). (**B**) Sedimentation equilibrium analysis of EcRHH–RcPutA. The curves represent global fit analysis of data from 0.2 mg/ml (circles), 0.5 mg/ml (squares) and 0.8 mg/ml (diamonds) EcRHH–RcPutA at 8000 rpm to an ideal single-species model yielding a 256 kDa species (theoretical 251 kDa). A vertical offset was applied to the residuals that are shown for each fit in the bottom panel. *M* values are reported at a 95% confidence interval.

EcRHH–RcPutA and RcPutA were first analysed by gel filtration chromatography. Figure 3(A) shows a striking difference between the two proteins. The elution profile of EcRHH–RcPutA estimates an *M* of approximately 315 kDa whereas RcPutA elutes at an apparent *M* of approximately 122 kDa consistent with that reported recently for RcPutA [29]. These results indicate that EcRHH–RcPutA forms a higher order oligomer.

EcRHH–RcPutA was also analysed by analytical ultracentrifugation. Sedimentation equilibrium experiments were performed at three protein concentrations. The equilibrium data for EcRHH–RcPutA (Figure 3B) were fit to an ideal single-species model, which yielded an *M* value of 256 kDa (Table 2). These results further show that EcRHH–RcPutA is a dimer (theoretic dimer *M* of 251 kDa). Thus, fusion of the EcPutA RHH domain induces dimerization of RcPutA.

**Table 2:** Structural parameters of EcRHH–RcPutA

	Parameters
*M* from AUC (kDa)*	256
*M* from SAXS (kDa)	220
Guinier *R*_g_ (Å)	51±1
Real space *R*_g_ (Å)	53–56
*V*_c_ (Å^2^)	1187
Porod volume (Å^3^)	383000
Oligomeric state	dimer

The *M* estimated from equilibrium analytical ultracentrifugation. The *M* estimated from *M*=*V*_c_^2^*R*_G_^−1^/0.1231.

### SAXS analysis of EcRHH–RcPutA

**Figure 4:**
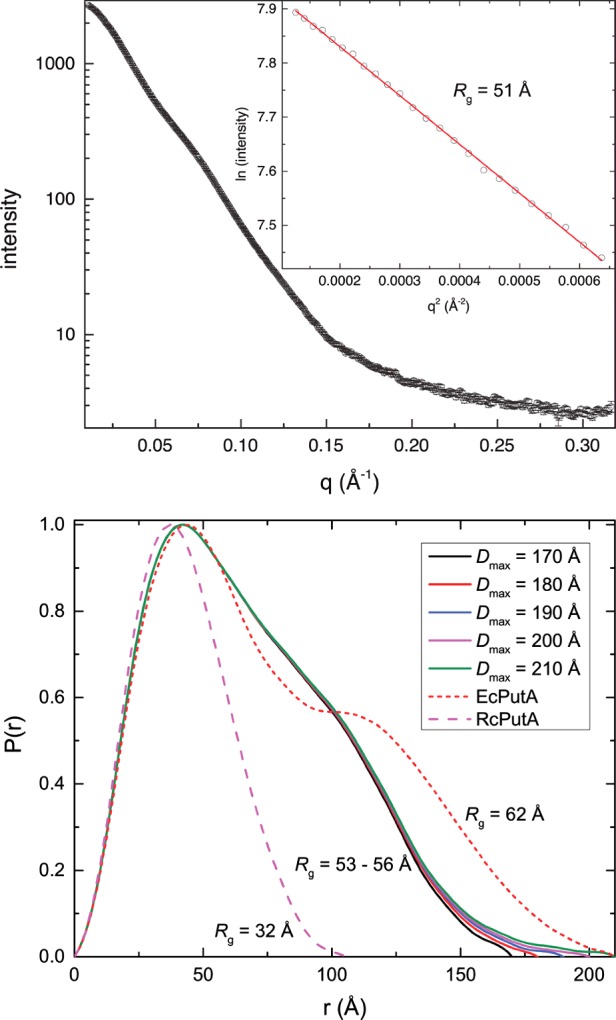
SAXS analysis of EcRHH–RcPutA (**A**) Experimental SAXS curve and Guinier analysis (inset). The Guinier plot spans the range of q*R*_g_=0.584–1.31. The linear fit of the Guinier plot has *R*^2^ of 0.9992. (**B**) P(*r*) curves for EcRHH–RcPutA for assumed *D*_max_ values of 170–210 Å. The P(*r*) curves for RcPutA [29] and EcPutA [46] are shown for reference.

EcRHH–RcPutA was analysed by SAXS, which can determine solution structural properties of proteins such the *R*_g_, *D*_max_, *M* and molecular shape envelope [47,48]. A SAXS curve for EcRHH–RcPutA is shown in Figure 4(A). The Guinier plots calculated from data collected at three protein concentrations exhibit good linearity (Figure 4A) and yield *R*_g_ values of 49.5–51.9 with an average of *R*_g_=51±1 Å (Table 2). Calculations of the pair distribution function (P(*r*)) suggest *R*_g_ of 53–56 Å for assumed *D*_max_ of 170–210 Å (Figure 4B). For comparison, the *R*_g_ and *D*_max_ of RcPutA are only 32 Å and 107 Å respectively [29], whereas those of full-length EcPutA are 63 Å and 205 Å [46]. The P(*r*) curve for EcRHH–RcPutA exhibits a major peak at *r*=42 Å with a shoulder on the high *r* side of the major peak. This distribution of interatomic vectors resembles that of EcPutA (Figure 4B) and is characteristic of a particle having two spatially separated lobes. In contrast, the P(*r*) curve for RcPutA is monomodal with maximum at *r*=37 Å (Figure 4B). These results show that EcRHH–RcPutA resembles EcPutA in terms of overall particle size and shape.

The *M*, and hence oligomeric state, of EcRHH–RcPutA was determined from the SAXS data using the *V*_c_ method [39] and Porod–Debye analysis [49] (Table 2). The SAXS *V*_c_ is estimated to be 1187 Å^2^, which corresponds to *M* of 220 kDa. This value is within 12% of the expected *M* of the EcRHH–RcPutA dimer (251 kDa) as described above. The estimated Porod volume is 383000 Å^3^. The assumption of a dimeric protein leads to a protein density of 1.1 g/ml, which is well within the characteristic range for proteins of 0.9–1.5 g/ml [49]. In contrast, the assumption of a monomeric or trimeric protein results in density values of 0.54 g/ml or 1.6 g/ml respectively, which are unrealistic for a compact folded protein. Thus, the SAXS data for EcRHH–RcPutA are consistent with a dimeric protein.

**Figure 5:**
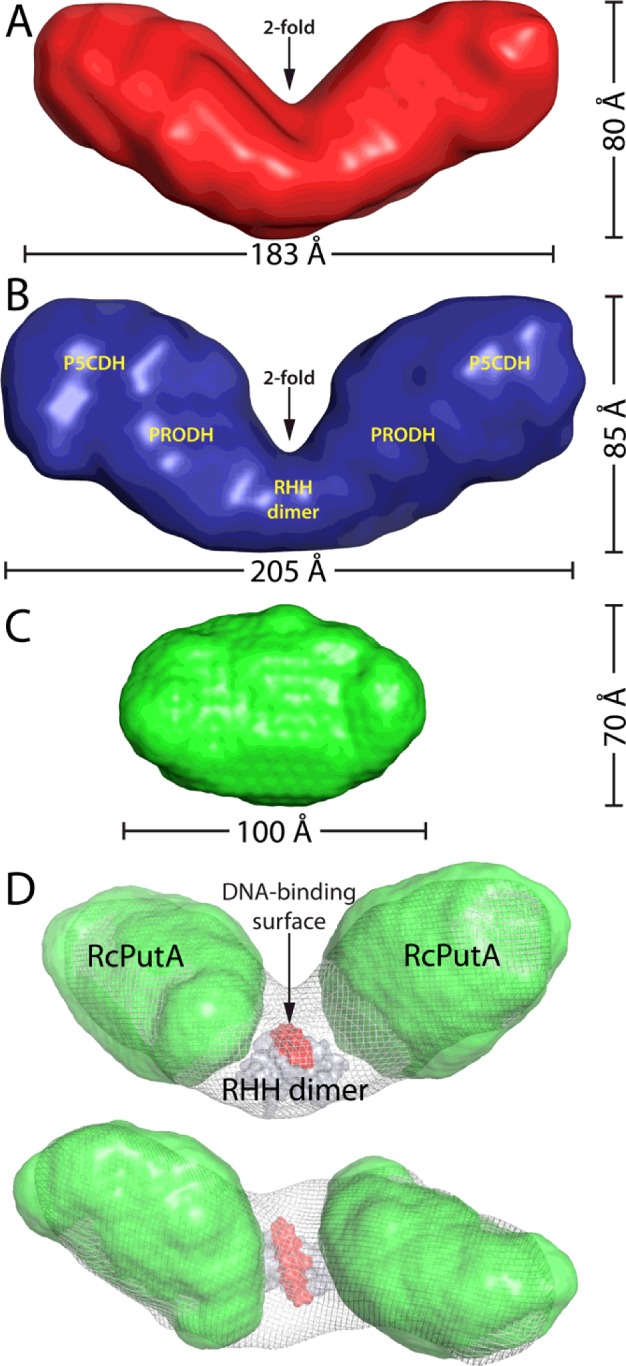
SAXS shape reconstructions (**A**) The envelope of EcRHH–RcPutA calculated from 32 independent GASBOR shape reconstruction calculations performed with the assumption of 2-fold symmetry. The normalized spatial discrepancy of the ensemble is 1.7±0.2. (**B**) The SAXS envelope of EcPutA. Reproduced from [46]: Singh, R.K., Larson, J.D., Zhu, W., Rambo, R.P., Hura, G.L., Becker, D.F. and Tanner, J.J. (2011) Small-angle X-ray scattering studies of the oligomeric state and quaternary structure of the trifunctional proline utilization A (PutA) flavoprotein from *Escherichia coli*. J. Biol. Chem. 286, 43144–43153. (**C**) The SAXS envelope of RcPutA. Reproduced from [29]: Luo, M., Christgen, S., Sanyal, N., Arentson, B.W., Becker, D.F. and Tanner, J.J. (2014) Evidence that the C-terminal domain of a Type B PutA protein contributes to aldehyde dehydrogenase activity and substrate channeling. Biochemistry 53, 5661–5673. (**D**) Orthogonal views of a model of the EcRHH–RcPutA dimer. The shape reconstructions of EcRHH–RcPutA and RcPutA are shown as white mesh and green surfaces respectively. The EcPutA RHH dimer (PDB code: 2GPE) is shown as a grey surface with the DNA-binding surface highlighted in red. This model was created by first fitting the RcPutA envelopes into the shape reconstruction of EcRHH–RcPutA using the Fit-in-Map option of chimaera [53]. Then, the EcPutA RHH dimer was manually docked into the remaining open space so that the molecular 2-fold axis of the RHH dimer is parallel to the 2-fold axis of the EcRHH–RcPutA envelope. This model is consistent with previous SAXS modelling studies of EcPutA [46].

SAXS shape reconstruction calculations were performed so that the molecular shape of EcRHH–RcPutA could be compared with those of RcPutA and EcPutA. The averaged and filtered model for EcRHH–RcPutA resulting from 32 independent calculations has a normalized spatial discrepancy of 1.7±0.2 with only two discarded models, which indicates that the ensemble is structurally homogeneous. EcRHH–RcPutA forms a V-shaped dimer in solution with dimensions of 183×80×69 Å (Figure 5A). EcPutA also forms a V-shaped dimer in solution and has dimensions comparable with those of EcRHH–RcPutA (205×85×55 Å, Figure 5B). Thus, EcRHH–RcPutA and EcPutA have the same basic size and shape. Note that this shape differs dramatically from the monomeric ellipsoid of RcPutA (Figure 5C).

Previous studies on EcPutA have shown that the catalytic domains reside in the two large outer lobes, whereas the RHH dimer occupies the connector between the two lobes (Figure 5B) [46]. This quaternary structure and domain arrangement appears to be present in EcRHH–RcPutA. For example, the outer lobes of the shape reconstruction of EcRHH–RcPutA are similar in size and shape to the SAXS envelope of RcPutA (Figure 5D). Furthermore, the connecting section between the two lobes is large enough to accommodate the RHH dimer of EcPutA (Figure 5D). It is concluded that fusion of the RHH domain endows RcPutA with the oligomeric state, quaternary structure and overall shape of EcPutA.

### DNA-binding and lipid-binding of EcRHH–RcPutA

**Figure 6:**
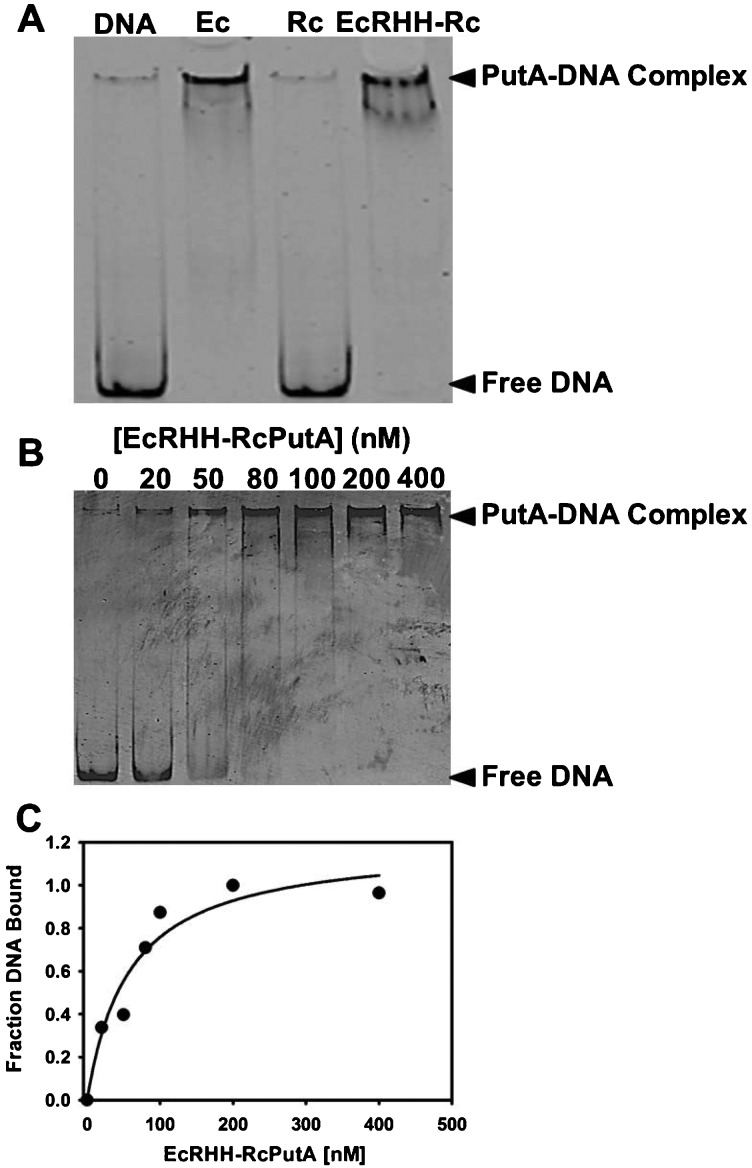
DNA-binding assays (**A**) Gel mobility shift assays of EcPutA (200 nM), RcPutA (200 nM) and EcRHH–RcPutA (200 nM) added to binding mixtures containing 3 nM of fluorescent labelled (IRdye-700 label) *put* control DNA (420 bp) and 100 μg/ml of nonspecific calf thymus DNA. Free DNA and PutA–DNA complexes were separated on a non-denaturing 4% polyacrylamide gel. (**B**) Representative gel mobility shift assay with increasing concentrations of EcRHH–RcPutA (0–400 nM) with fluorescently labelled *put* control DNA (3 nM). (**C**) Plot of EcRHH–RcPutA concentration compared with fraction of DNA bound from two independent gel-shift assays. Data were fit to eqn (1) to yield a dissociation constant (*K*_d_) of 58±20 nM.

From the analysis above, it appears EcRHH–RcPutA adopts a similar oligomeric state and spatial arrangement of the DNA-binding and catalytic domains as that of EcPutA. We next sought to determine if EcRHH–RcPutA exhibited the functional switching properties of EcPutA. The DNA-binding activities of EcPutA, RcPutA and EcRHH–RcPutA were compared using gel mobility shift DNA-binding assays. Figure 6(A) shows that RcPutA does not bind DNA, as expected, whereas EcRHH–RcPutA binds *put* control DNA similarly to EcPutA. A dissociation constant (*K*_d_) for EcRHH–RcPutA with *put* control DNA was determined by varying EcRHH–RcPutA concentration in the binding assays as shown in Figure 6(B). Figure 6(C) shows a plot of the fraction of bound DNA compared with EcRHH–RcPutA concentration fit to eqn (1) (*n*=1.2), from which a *K*_d_ value of 58±20 nM was estimated. This value is nearly the same as that determined previously for EcPutA (*K*_d_=45 nM) [17].

**Figure 7:**
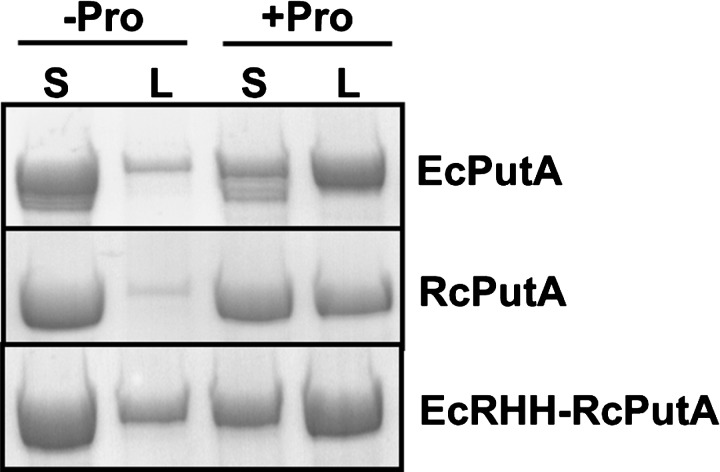
Lipid pull-down assays EcPutA, RcPutA and EcRHH–RcPutA (0.3 mg/ml each) were incubated with or without proline in HEPES buffer (pH 7.5, 150 mM NaCl) with freshly prepared *E. coli* polar lipid vesicles (0.8 mg/ml) for 1 h at room temperature. Following incubation, soluble and lipid fractions were separated by Airfuge ultracentrifugation and analysed via SDS/PAGE.

Lipid pull-down assays were used to test whether the membrane binding properties of RcPutA and EcRHH–RcPutA are regulated by proline reduction of the flavin cofactor as observed for EcPutA [22,26]. RcPutA, EcRHH–RcPutA and EcPutA were incubated with *E. coli* polar lipid vesicles in the absence and presence of 50 mM proline. After incubation for 1 h, the soluble and lipid fractions were separated by centrifugation and analysed by SDS/PAGE to monitor PutA partitioning in the soluble and lipid fractions. Figure 7 clearly shows that in the absence of proline (i.e. oxidized state) RcPutA and EcRHH–RcPutA are mainly in the soluble fraction similar to EcPutA. In the presence of proline, RcPutA and EcRHH–RcPutA significantly partition into the lipid fraction, indicating that membrane interactions are favoured in a reducing environment as seen previously with EcPutA [26]. These results indicate that RcPutA- and EcRHH–RcPutA-membrane associations are redox regulated similarly to that of EcPutA [19].

## DISSCUSSION

Fusing the EcRHH domain to RcPutA generated a stable EcRHH–RcPutA dimer providing evidence that dimerization in trifunctional PutAs is mediated through the DNA-binding domain [24,46]. Kinetic assays showed that the dimeric structure of EcRHH–RcPutA does not affect the catalytic properties of RcPutA. The PRODH activity of EcRHH–RcPutA is similar to RcPutA whereas the progress curve of the overall PRODH–P5CDH reaction of EcRHH–RcPutA has no observable lag phase consistent with a substrate channelling mechanism as shown recently for RcPutA [29]. The SAXS analysis shows that although EcRHH–RcPutA and EcPutA have the same shape and quaternary structure, the chimaera is approximately 20 Å shorter in one dimension. As a result, the catalytic lobes are closer together in the EcRHH–RcPutA than in EcPutA. This was of interest to us as the spacing of the catalytic lobes must be wide enough to allow the RHH domain to interact with DNA. In EcRHH–RcPutA, the spacing is narrower relative to EcPutA, possibly restricting the accessibility of the RHH domain to DNA. EcRHH–RcPutA, however, exhibits DNA-binding similar to EcPutA indicating that the spatial separation of the catalytic lobes is adequate for the DNA binding activity of EcRHH–RcPutA. The lipid pull-down assays show proline enhances EcRHH–RcPutA membrane binding consistent with the redox switching mechanism of EcPutA. These results, along with the DNA binding activity, suggest that RcPutA has been successfully converted into a trifunctional PutA protein by adding the EcRHH domain.

Our findings suggest the trifunctionality of type C PutA derives from a dimeric structure anchored by a RHH domain that is sandwiched between two catalytic lobes. The spacing of the catalytic lobes is likely critical to enable the RHH domain to access DNA. Although the oligomeric state and catalytic properties of RcPutA were previously determined, the membrane binding properties RcPutA were unstudied [29]. In the present study, we showed that RcPutA displays enhanced membrane binding in the presence of proline. Heretofore, this property was thought to be exclusive to trifunctional PutAs. For example, the membrane-binding affinity of the type A PutA from *B. japonicum* was shown to be insensitive to proline [50]. The physiological rationale for regulating RcPutA membrane interactions is not clear, as RcPutA does not have a transcriptional repressor function. In *R. capsulatus*, *putA* expression is activated in response to proline by the PRODH activator, PutR [51]. PutR contains a helix-turn-helix motif and is a member of the Lrp/AsnC family of DNA binding proteins [51]. Nevertheless, our results for RcPutA indicate that type B PutAs contain at least some of the structural elements that are responsible for functional switching.

**Figure 8:**
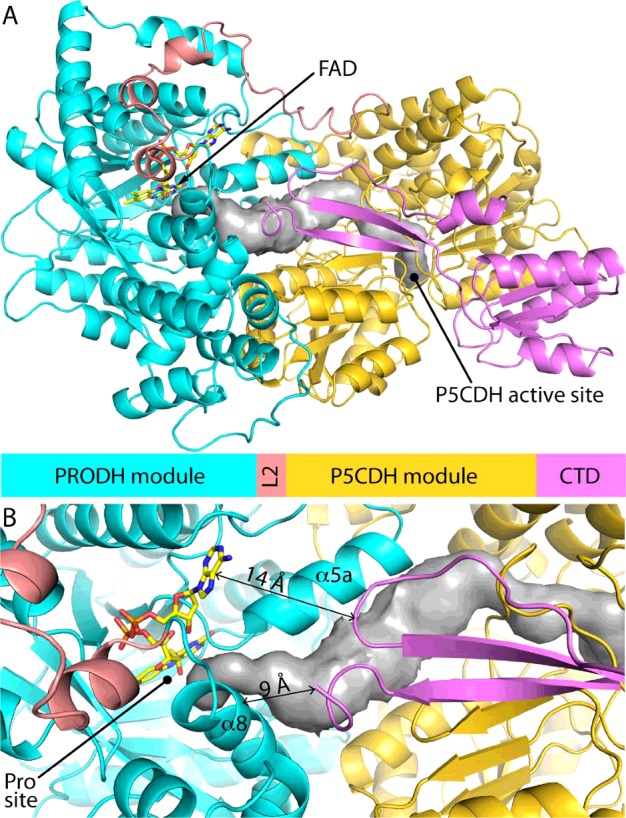
Model of RcPutA from SAXS rigid body modelling [29] (**A**) Model of RcPutA highlighting domain architecture and locations of the active sites. The grey surface represents the substrate-channelling tunnel. (**B**) Close-up view showing the predicted spatial proximity of the PRODH active site and CTD β-flap.

The CTD may be an element of the functional switching apparatus, since this domain is absent in type A PutAs and present in types B and C PutAs. Recent studies of RcPutA suggested that the CTD is a structural domain that couples the PRODH and P5CDH domains, and facilitates P5CDH activity and substrate channelling [29]. The data presented here furthermore implicate the CTD in functional switching, since RcPutA-membrane association was shown to be redox regulated, similar to trifunctional PutAs, and this domain is missing in non-switching type A PutAs. This role is consistent with our model of RcPutA showing that the  β-flap of the CTD approaches the second sphere of the PRODH active site, contacting helices α8 and α5a of the PRODH domain (Figure 8). This spatial relationship is potentially significant because α8 contains several conserved residues that bind the substrate proline and has been shown to shift by several Å upon flavin reduction [31,52]. Furthermore, α5a directly contacts the FAD and is a PutA-specific secondary structural element not found in monofunctional PRODHs (Figure 8B). Thus, we suggest that the CTD may be appropriately positioned to sense and transduce conformational changes in the PRODH domain induced by flavin reduction. Delineation of the membrane-binding region of PutAs in future studies will be necessary to understand the potential role of the CTD in functional switching and to identify structural elements involved in reductive activation of PutA-lipid binding.

## Abbreviations

CoQ1: coenzyme Q1
CTD: C-terminal domain
*D*_max_: maximum particle dimension
EcPutA: *Escherichia coli* PutA
EcRHH: ribbon–helix–helix domain of EcPutA
EcRHH–RcPutA: chimaera consisting of the RHH domain of EcPutA fused to the N-terminus of RcPutA
GSA: glutamate-γ-semialdehyde
*M*: molecular mass
P5C: Δ^1^-pyrroline-5-carboxylate
P5CDH: Δ^1^-pyrroline-5-carboxylate dehydrogenase
PRODH: proline dehydrogenase
PutA: proline utilization A
RcPutA: proline utilization A from *Rhodobacter capsulatus*
*R*_g_: radius of gyration
RHH: ribbon–helix–helix
THP: tris(3-hydroxypropyl)phosphine
*V*_c_: volume of correlation
